# City- and county-level spatio-temporal energy consumption and efficiency datasets for China from 1997 to 2017

**DOI:** 10.1038/s41597-022-01240-6

**Published:** 2022-03-24

**Authors:** Jiandong Chen, Jialu Liu, Jie Qi, Ming Gao, Shulei Cheng, Ke Li, Chong Xu

**Affiliations:** 1 School of Public Administration, Southwestern University of Finance and Economics, Chengdu, Sichuan 611130 China; 2 School of Statistics, Southwestern University of Finance and Economics, Chengdu, Sichuan 611130 China

**Keywords:** Energy economics, Climate-change mitigation

## Abstract

Understanding the evolution of energy consumption and efficiency in China would contribute to assessing the effectiveness of the government’s energy policies and the feasibility of meeting its international commitments. However, sub-national energy consumption and efficiency data have not been published for China, hindering the identification of drivers of differences in energy consumption and efficiency, and implementation of differentiated energy policies between cities and counties. This study estimated the energy consumption of 336 cities and 2,735 counties in China by combining Defense Meteorological Satellite Program/Operational Line-scan System (DMSP/OLS) and Suomi National Polar-Orbiting Partnership/Visible Infrared Imaging Radiometer Suite (NPP/VIIRS) satellite nighttime light data using particle swarm optimization-back propagation (PSO-BP). The energy efficiency of these cities and counties was measured using energy consumption per unit GDP and data envelopment analysis (DEA). These data can facilitate further research on energy consumption and efficiency issues at the city and county levels in China. The developed estimation methods can also be used in other developing countries and regions where official energy statistics are limited.

## Abstract (summary)

**Table:** 

Measurement(s)	fossil fuel
Technology Type(s)	machine learning

## Background & Summary

China has been the world’s highest energy consumer since 2007, followed by the United States^1–3^. Optimizing the energy mix and improving energy efficiency is crucial for China itself and the world response to climate change. Accurate measurement of China’s energy consumption and efficiency, particularly at the sub-national level, is essential to accelerate the achievement of the energy transition and energy efficiency improvement goals.

China’s national energy consumption data is published by the Chinese National Bureau of Statistics (CNBS)^4–6^, International Energy Agency (IEA)^7–9^, British Petroleum (BP)^10–12^, Energy Information Administration (EIA)^13–15^, World Bank^16,17^, and other international organizations, while China’s provincial-level energy consumption data is mainly published by the CNBS^18–20^. These widely accepted energy consumption data have substantially contributed to research on international energy consumption comparisons^13,15,21^, overall and inter-provincial energy consumption drivers^22–24^, and socioeconomic effects of overall and inter-provincial energy consumption in China^5,25,26^.

The availability of China’s national and provincial-level energy consumption data also indirectly provides key support for energy efficiency studies. Energy efficiency not only reflects the coupling relationship between energy consumption and economic development, but also implies the energy policy appeal to maximize economic output based on energy input. To deal with climate change under the sustainable development goals, it is necessary to drive sustained economic development with as little energy consumption as possible. The measurement of energy efficiency can be classified into three categories:

First, the indicator measurement analysis (IMA) is used to measure energy efficiency by energy consumption per unit GDP^4^. The advantages of this measurement are mainly reflected in its low requirements for other socioeconomic data, simple calculation and easy to be regarded as a policy target^17^. Most countries, including China, have set reducing energy consumption per unit GDP as a government policy target to tackle climate change^17^. However, it cannot reveal the improvement path of energy efficiency.

Second, the non-parametric method, in which energy, capital and labor are regarded as inputs, GDP and pollutants are regarded as expected output and undesirable output respectively, is applied to obtain energy efficiency. Energy efficiency means that reducing energy consumption as much as possible in exchange for increased economic output and fewer pollutants^24^. The closer the actual output increase and pollutant reduction in exchange for the reduction of energy consumption are to the theoretical potential output increase and pollutant reduction, that is, the closer the actual energy performance is to the potential energy performance, the higher the energy efficiency will be. The data envelopment analysis (DEA) is the most widely used non-parametric method^24^. The advantages of this measurement are mainly reflected in the convenience of locking the drivers of energy efficiency improvement, the ease of comparison of the relative energy efficiency between regions, and the need for no prior setting of the estimation function. However, it not only requires more socioeconomic data except energy consumption, but also is relatively complicated to calculate.

Third, the parameter method, which takes energy, capital and labor as independent variables and GDP as dependent variable, is chosen to estimate energy efficiency^24^. The stochastic frontier analysis (SFA) is the most widely used parameter method^27^. However, because of relying on the prior setting of the regression function and requiring more socioeconomic data except energy consumption, this measurement is relatively few adopted.

Although research on energy efficiency has practical values, unlike energy consumption data, neither Chinese officials nor other international organizations have published energy efficiency data. This is mainly because energy efficiency data is the use of energy consumption data. If Chinese officials and other international organizations publish energy consumption data, scholars and institutions can use the above methods to obtain energy efficiency data. However, the widely accepted energy efficiency data only focus on the national and provincial levels, because the energy consumption data published by the CNBS and other international organizations is at national and provincial levels^24,27^.

Socioeconomic development and energy consumption vary considerably between cities and counties in sub-provincial China^27–29^. For example, in 2010, energy consumption was approximately 30 times higher in Tangshan than in Suqian^30^. Meanwhile, even cities with similar scales of energy consumption have different levels of economic development. For example, in 2010, the energy consumption of Wenzhou is similar to that of Jiayuguan, the GDP of the former is nearly 15 times that of the latter^30^. However, official data are unavailable on city-and-county-level spatio-temporal energy consumption and efficiency in China. This hinders microscopic-level research on the drivers of energy consumption and assessment of energy efficiency in China. Moreover, this limits central and provincial governments in setting differentiated energy efficiency targets and city and county governments in adopting targeted energy efficiency improvement initiatives. Therefore, numerous studies have recently attempted to overcome these research constraints. These studies can be broadly classified into the following three categories.

First, city-level energy consumption data are collated from provincial and city statistical yearbooks^31–33^. Although the data from these studies are highly reliable as they are published by official agencies, they lack annual energy consumption data for all cities, and the energy types counted by some city statistics departments vary. This is mainly because the lower the government level, the less efficient their data disclosure^34^ and the weaker the accounting capacity of energy statistics departments, particularly in developing cities.

Second, city-and-county-level energy consumption surveys are conducted to obtain energy consumption data at these levels, and at the enterprise and household levels^35–38^. The data obtained from these surveys are more microscopic, accurate, and complete; however, annual energy consumption data cannot be obtained for all cities and counties, and the energy consumption status and change characteristics for both cities and counties cannot be directly generalized. This is primarily due to the high labor and financial resources required to conduct these surveys^35^, the difficulty of including all cities and counties, and the difficulty of conducting annual surveys.

Third, satellite data and machine learning methods are used to determine city-level CO_2_ emissions related to energy consumption. For example, Chen *et al*.^39,40^ employed particle swarm optimization-back propagation (PSO-BP) to construct quantitative relationships between provincial nighttime light data and statistical provincial CO_2_ emission data in China, and applied top-down data inversion to derive CO_2_ emission data for all cities and counties in China, by using the total lighting brightness in these cities and counties as weights. Yue *et al*.^41^ used econometric regression and two nighttime light data to obtain a 1-km resolution energy consumption data on the regional scale for China. Although these studies do not provide energy consumption data at the city and county levels, they provide a useful way to obtain annual energy consumption data for cities and counties in China.

Our study filled this gap through inversion of satellite data and machine learning methods to obtain spatio-temporal energy consumption data for 336 cities and 2,735 counties in China from 1997 to 2017 by combining Defense Meteorological Satellite Program/Operational Line-scan System (DMSP/OLS) and Suomi National Polar-Orbiting Partnership/Visible Infrared Imaging Radiometer Suite (NPP/VIIRS) satellite nighttime light datasets using PSO-BP. Based on these energy consumption and related socioeconomic data, the study also provides spatio-temporal energy efficiency data at the city and county levels by using the energy consumption per unit GDP and the ratio of actual to potential energy performance in the non-radial directional distance function (NDDF) derived from DEA. The proposed methodology and datasets can be widely used in energy consumption and efficiency studies at the city and county levels in China, and can provide a reference for other developing countries and regions with limited energy statistics to analyze their sub-national energy consumption and efficiency.

## Methods

### Data scopes

In this study, the measured energy consumption and efficiency datasets were based on city and county levels in China. Cities and counties are the grassroot administrative regions in China and the basic units in the implementation of energy policies by the Chinese government^23,42,43^. Energy consumption at city-and-county-level excludes the production side of the jurisdiction and the energy consumption transferred across administrative regions.

Our study provides data on energy consumption for 336 cities (including 332 prefecture-level cities and 4 municipalities) and 2,735 counties in mainland China from 1997 to 2017 (excluding Hong Kong, Macau, Taiwan, and Tibet) based on the accessibility of the identifiable satellite nighttime light data with a certain resolution, which covers approximately 87% of the land area, over 90% of the population, and 90% of the GDP. The definitions of cities and counties are published in 2010 by the Ministry of Civil Affairs of China. Considering the names of some cities and counties have changed after 2010, our study used their names of 2010 to keep consistency. More detailed explanation was shown in the data files^44^.

Our study also provides two energy efficiency datasets: First, energy efficiency data was calculated by energy consumption per unit GDP, which covered 336 cities and 2,489 counties (1997–2017). It includes fewer cities and counties in some years, due to the lack of GDP data. Second, energy efficiency data was calculated by the ratio of actual to potential energy performance (also known as energy efficiency performance index, EEPI) using the NDDF of DEA, which covered 189 cities (2003–2016). It includes fewer years and cities, and none counties, mainly due to the lack of basic socioeconomic data considered as input variables of the NDDF, for example, fixed asset investment and fixed asset investment price index.

### Matching nighttime light data from two types of satellites

The city-and-county-level energy consumption data in this dataset were inferred from two satellite nighttime light datasets: the DMSP/OLS and the NPP/VIIRS^45^. The DMSP/OLS data are derived from the OLS scanners from 1992 to 2013^45,46^, and NPP/VIIRS data from the VIIRS scanners from 2012^47^. Although the above two accessible nighttime light data have the advantages of long time span and wide space coverage, and are widely accepted and applied, they cannot be directly mixed together. It is mainly because the two nighttime light images belong to different sensors, different time and space of nighttime light image collection and differences in pixel levels. For example, the inconsistencies in the acquisition time and cloud cover of nighttime light images in 2013 resulted in a large gap between DMSP/OLS and NPP/VIIRS images at the pixel level. Moreover, the two nighttime light data also have the different discontinuities and saturation levels, and the differences in spillover and white noise. Thus, to obtain a long and continuous nighttime light data (including those before and after 2013), we spliced the two satellite nighttime light datasets following the procedures adopted by Liu *et al*.^48^, Lv *et al*.^49^, and Chen *et al*.^39^.

First, inter-calibration^50^, radiometric calibration^51^, intra-annual composition^52,53^ and inter-annual series correction methods^53^ were adopted for correcting DMSP/OLS data to eliminate pixel under-saturation and spillover as well as discontinuities, incomparability, and instability. Inter-calibration method is an on-board calibration and additional processes on the annual composites of nighttime light data. Considering both satellite sensors transferring nighttime light data in the same year, such as F14 and F15 in 2001, we applied the intra-annual composition and to improve the stability of lit pixels, as follows:

1 $$D{N}_{\left(n,i\right)}=\left\{\begin{array}{c}0,D{N}_{\left(n,i\right)}^{a}=0\;and\;D{N}_{\left(n,i\right)}^{b}=0\\ \frac{D{N}_{\left(n,i\right)}^{a}+D{N}_{\left(n,i\right)}^{b}}{2},else\end{array}\right.$$

where $D{N}_{\left(n,i\right)}$ represents the digital number (DN) values of lit pixel *i* from two types of satellite sensors in year *n*.

Radiometric calibration method is used for eliminating the differences between nighttime light data in the same year from two satellites when conducting comparative time series analysis. We applied the invariant region method to inter-calibrate DMSP/OLS images, which is consistent with Wu *et al*.^51^. It has better inter-calibration accuracy. Power function form was chosen and the global radiance calibrated nighttime light (RCNTL) in 2006 was treated as a reference image. The estimated parameters are shown in Table 1.

**Table 1:** Coefficients of the power function for nighttime light data (RCNTL in 2006 as a reference image).

Satellite	Year	*d*	*e*	*R*^2^
F10	1992	1.158	1.17	0.7955
F10	1993	2.105	0.896	0.8103
F10	1994	1.264	1.127	0.9093
F12	1994	3.074	0.765	0.8909
F12	1995	1.300	1.073	0.9151
F12	1996	1.491	1.029	0.9018
F12	1997	0.947	1.192	0.8952
F12	1998	0.884	1.167	0.8220
F12	1999	1.473	1.008	0.8657
F14	1997	2.264	0.97	0.8619
F14	1998	1.485	1.077	0.8629
F14	1999	1.454	1.137	0.8395
F14	2000	1.282	1.108	0.8628
F14	2001	1.179	1.129	0.8978
F14	2002	1.598	0.987	0.9242
F14	2003	1.394	1.038	0.8729
F15	2000	1.196	1.075	0.8514
F15	2001	1.341	1.033	0.8423
F15	2002	0.889	1.151	0.8411
F15	2003	1.824	0.974	0.8427
F15	2004	1.236	1.07	0.8838
F15	2005	1.764	0.937	0.8564
F15	2006	1.225	1.061	0.9066
F15	2007	1.324	1.067	0.9103
F16	2004	0.807	1.143	0.8790
F16	2005	1.058	1.119	0.9191
F16	2006	1.005	1.117	0.9352
F16	2007	0.965	1.109	0.8911
F16	2008	0.855	1.108	0.9480
F16	2009	0.543	1.175	0.9176
F18	2010	0.273	1.304	0.8914
F18	2011	0.694	1.09	0.8351
F18	2012	0.474	1.179	0.8743
F18	2013	0.418	1.153	0.8501

Note: *d*, *e*, and *R*^2^ represent the constant term, index, and coefficient of determination, respectively.

Inter-annual series correction method is used for stabilizing the inter-annual variability of the same satellite. According to Hu & Huang^53^, it was performed by assuming that the DN value of each lit pixel would not decrease over time with the economic development, as follows:

2 $$D{N}_{\left(n,i\right)}=\left\{\begin{array}{c}D{N}_{\left(n-1,i\right)},D{N}_{\left(n-1,i\right)}\ge D{N}_{\left(n,i\right)}\\ D{N}_{\left(n,i\right)},else\end{array}\right.$$

**Fig. 1:**
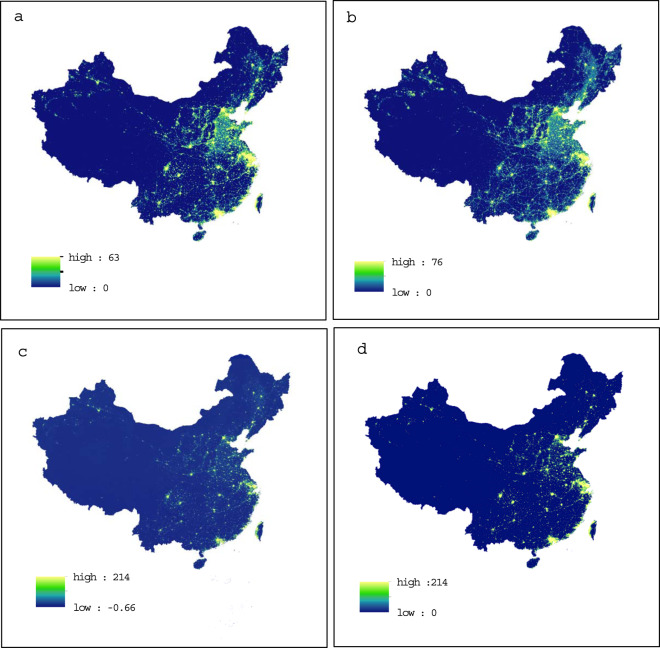
Differences before and after pre-processing of DMSP/OLS and NPP/VIIRS data. (**a**) The orginal DMSP/OLS images in 2013 before pre-processing, (**b**) the DMSP/OLS images in 2013 after pre-processing, (**c**) the original NPP/VIIRS images in 2017 before pre-processing, (**d**) the NPP/VIIRS images in 2017 after pre-processing.

Moreover, as the higher intensity summer light in high latitudes can cause increased interference with the accuracy of the NPP/VIIRS data^49^, the monthly NPP/VIIRS data for June, July, and August were excluded and the annual data was synthesized as an arithmetic mean based on the nighttime light data for the remaining nine months. To avoid the influence of deleting abnormal nighttime light data of the above three months on the accuracy of matching, noise pollution was removed in the process of converting NPP/VIIRS data into NPP/VIIRS data, which is consistent with Lv *et al*.^49^. Accordingly, we used a Gaussian low-pass filter with a 5 × 5 pixel window to smooth the NPP/VIIRS annual data and reduce spatial variability^54^ for better matching with the DMSP/OLS annual data. The Gaussian low-pass filter *δ* was set to 1.75^54,55^ and the negative value was replaced with zero. To further reduce the effect of white noise, a threshold $0.3nWc{m}^{-2}s{r}^{-1}$ was set in the annual image, and data smaller than this threshold were excluded^52^. Fig. 1 reported the differences by comparing the DMSP/OLS images in 2013 and the NPP/VIIRS images in 2017 before and after pre-processing, respectively. By comparing Fig. 1a and 1b, the processed images have solved the saturation issue of the original images that all DN values of these saturated pixels were 63. Meanwhile, compared to Fig. 1c, the processed images in Fig. 1d have eliminated negative values and maintained better matching with the DMSP/OLS images in terms of the distribution of nighttime light values.

Second, PSO-BP was used to match the DMSP/OLS, which has differences in sensors and resolution, with the NPP/VIIRS satellite nighttime lighting dataset. The PSO, proposed by Kennedy & Eberhart^56^, is an intelligent global optimization algorithm based on the laws of collaborative mechanisms in bird populations. Each particle in the swarm represents a possible solution to the problem, and each particle corresponds to a fitness value whose position and velocity will be determined by the optimal position^57^. After each position is updated, its fitness value is recalculated, and its position and velocity are updated again, so that all particles reach the optimal solution after repeated iterations^57^. Rumelhart *et al*.^58^ developed a multilayer feedforward neural network algorithm with a three-layer network topology, also known as BP network algorithm, which included an input layer, an output layer, and an implicit layer. The number of neurons in the implicit layer and the transfer function between the nodes are related to the specific object under study, and there is no unified principle for selecting them^59,60^; however, they can generate local data at local extremes and cause training failures^61^. The BP algorithm is a local algorithm with good performance in constructing regressions and obtaining local optimum optimistic results, however, data at local extremes and training failures may occur. The PSO algorithm is a global algorithm which has the potential to explore the global optimistic results^62^. The combination of these two algorithms helps to improve the computational effectiveness^63^. Ismail *et al*.^64^, Mohamad *et al*.^63^, Lee & Cheng^57^, and Wang *et al*.^28^ have successively combined these two algorithms and proposed the PSO-BP neural network algorithm. This improved algorithm can overcome the shortcomings of the traditional BP neural network, such as poor learning stability, low reliability, and easily attained local minima. The specific steps are as follows^63,65^:

To build the neural network model, we set the county-level NPP/VIIRS pixel averages for 2013 as input parameters, and derived the central geographical coordinates of each county in China using the minimum boundary method, following Chen *et al*.^40^. By applying the Lambert projection and the ArcGIS zone statistics, we obtained the area of each county using Arcmap 10.5, and used the abovementioned coordinate data as well as the area data of each county as supplementary data parameters. This approach reduces matching errors and improves the integration accuracy of the two sets of satellite nighttime lighting data. We logarithmized the input parameters according to Li *et al*.^54^. When setting the output parameters, the 2013 county DMSP/OLS pixel averages are used as the output parameters.

The computational program developed by Ismail *et al*.^64^ and Mohamad *et al*.^63^ was used as a reference for the inter-calibration of the night-light data from DMSP/OLS and NPP/VIIRS. The number of layers of BP neural network is set to three, in which the hidden layer is set to five nodes, the maximum number of iterations is set to 50, and the population size is 20^54^. There are 2,735 counties in the Chinese mainland; therefore, we randomly selected 2,000 counties as our training set and 735 counties as our test set. However, even though the algorithm results obtained a high coefficient of determination, a large amount of data might also cause problems such as anomalous value, fluctuations, and jumps and faults in the conversion process. Thus, continuous satellite nighttime light data might not be obtained, leading to large errors in the estimated energy consumption data. To reduce this error, we further converted NPP/VIIRS data from 2013 to DMSP/OLS data, and calculated the annual growth of NPP/VIIRS data from 2013 to 2017 after conversion. For example, the annual growth of NPP/VIIRS data for 2014 and the existing DMSP/OLS data for 2013 provided the simulated 2014 DMSP/OLS data. Then, we can calculate the smooth DMSP/OLS data obtained from the 2014–2017 simulation^40^, as shown in the following equations.

3 $$\Delta D{V}_{i,t}=D{V}_{i,t}-D{V}_{i,t-1}$$

4 $${D}_{i,t}={D}_{i,t-1}+{\Delta }D{V}_{i,t}$$

where ${D}_{i,t}$ is the nighttime light data of DMSP/OLS, $D{V}_{i,t}$ is the DMSP/OLS nighttime light data transformed by NPP/VIIRS nighttime light data, $\Delta D{V}_{i,t}$ is the annual increment of NPP/VIIRS nighttime light data after transforming, *i* represent counties (*i*=1, 2, 3,…, 2735), and *t* represent years ($t=2014,2015,2016,2017$).

### Estimation of satellite-based energy consumption at the city and county levels

For the inversion of county-level energy consumption data, the provincial energy balance sheet data published in the *China Energy Statistics Yearbook* and the previously mentioned PSO-BP neural network algorithm were used to establish the quantitative relationship between provincial energy consumption data and provincial nighttime light data. The values of sum of the DN (SDN) value, and dummy variables of identity and year were selected as input parameters, and the provincial energy consumption were selected as output parameters. The reason why dummy variables of identity and year were selected in addition to the SDN values is to control the influence of province differences that do not change with time and time differences that do not change with time on provincial energy consumption. For example, although the DN values of two provinces are the same in a certain year, the different levels of energy consumption may still occur because of other parameters that have not been observed and vary with provinces. Similarly, although a province has the same DN value in two years, it may still have different energy consumption due to other parameters that have not been observed over time. The total energy consumption sample was 630 (21 years × 30 provinces), of which 400 samples were randomly selected as the training set, and the remaining 230 samples were selected as the test set. County energy consumption data^66–68^ were obtained using the top-down method and a DN value-based weighted-average strategy. Finally, 2,735 county-level energy consumption data were aggregated to obtain 336 city-level energy consumption data. The equation is as follows:

5 $$C{E}_{i,t}=\frac{SD{N}_{i,t}}{SD{N}_{j,t}}P{E}_{i,t}$$

where $C{E}_{i,t}$ represents county energy consumption, $SD{N}_{i,t}$ represents county lighting brightness, $SD{N}_{j,t}$ represents province lighting brightness, $P{E}_{i,t}$ represents province energy consumption, *i* represents county ($i=1,2,3,\ldots ,2735$), *j* represents province ($j=1,2,3,\ldots ,30$), and *t* represents year ($t=1997,1998,1999,\ldots ,2017$).

### Calculation of energy efficiency based on two methods

This dataset uses the DEA and the IMA to measure the energy efficiency data at the city and county levels in China. DEA is a non-parametric method widely used to measure efficiency^69–71^. Researchers worldwide have adopted and constantly improved this approach in their studies on energy efficiency^72–75^. The energy efficiency can be calculated using this method without setting the production function between input and output, which could be regarded as its advantage. According to the studies by Chen *et al*.^76^, Jebali *et al*.^77^, Wang *et al*.^78^, and Wu *et al*.^79^, we selected labor, capital, and energy as input variables; GDP as expected output variable; and particulate matter (PM_2.5_) as undesirable output variable.

We chose the NDDF with an undesired output as the non-parametric functions. This is because, unlike the traditional Sheppard distance function, the NDDF with undesirable output considers both the expected output and the undesirable output (usually the pollutant), and it does not require price-specific data. Moreover, compared with the traditional directional distance function, NDDF with undesired output can avoid the problem of overestimating efficiency due to the existence of slacks. Furthermore, NDDF can calculate the inefficiency value of each input and output factor^80–82^. Therefore, the use of NDDF is widely accepted in measuring energy efficiency^83–85^. By referring to the studies by Zhou *et al*.^86^, Zhang *et al*.^87^, and Lin & Chen^88^, we set the collection of weights for energy efficiency *EEPI* as $\left(0,0,0,1/3,1/3,1/3\right)$ and the set of directions as $\left(0,0,0,0,0,0\right)$, details as follows:

6 $$\mathop{\to }\limits_{{D}_{E}}\left(K,L,E,Y,B;g\right)={\rm{\max }}\;{w}_{E}\;{\beta }_{E}+{w}_{Y}{\beta }_{Y}+{w}_{B}\;{\beta }_{B}$$

7 $$\begin{array}{c}s.t.\mathop{\sum }\limits_{n=1}^{N}{Z}_{n}{K}_{n}\le K\\ \mathop{\sum }\limits_{n=1}^{N}{Z}_{n}{L}_{n}\le L\\ \mathop{\sum }\limits_{n=1}^{N}{Z}_{n}{E}_{n}\le E-{\beta }_{E}\,{g}_{E}\\ \mathop{\sum }\limits_{n=1}^{N}{Z}_{n}{G}_{n}\ge Y+{\beta }_{Y}\,{g}_{Y}\\ \mathop{\sum }\limits_{n=1}^{N}{Z}_{n}{B}_{n}=B-{\beta }_{B}\,{g}_{B}\end{array}$$

8 $${Z}_{n}\ge 0,n=1,2,\ldots ,N$$

9 $${\beta }_{E},{\beta }_{Y},{\beta }_{B}\ge 0$$

10 $$EEPI=\frac{1/2\left[\left(1-{\beta }_{E}^{\ast }\right)+\left(1-{\beta }_{B}^{\ast }\right)\right]}{1+{\beta }_{Y}^{\ast }}=\frac{1-1/2\left({\beta }_{E}^{\ast }+{\beta }_{B}^{\ast }\right)}{1+{\beta }_{Y}^{\ast }}$$

where $\mathop{\to }\limits_{{D}_{E}}(\cdot )$ represents the directional distance function; and *K* represents the capital stock, which is calculated by adopting perpetual inventory method based on the fixed asset investment data in the *China City Statistical Yearbook*: ${K}_{t}={I}_{t}+\left(1-{\alpha }_{t}\right){K}_{t-1}$, where ${I}_{t}$ is the current fixed asset investment, and ${\alpha }_{t}$ is the depreciation rate of fixed assets. Referring to the set of Meng *et al*.^89^, ${\alpha }_{t}$ is assumed to be 9.6%, and ${K}_{t-1}$ is the capital stock of the previous period. After using the fixed asset investment price index to deal with the fixed asset investment, the capital stock at the beginning of the period is set as ${K}_{2003}={I}_{2003}/\left({\alpha }_{t}+{p}_{n}\right)$ according to Li^90^, where ${K}_{2003}$ is the capital stock at the beginning of 2003, ${I}_{2003}$ represents the value of fixed asset investment in 2003 after adjusting for prices, and ${p}_{n}$ is the geometric mean of the growth rate of real fixed asset investment. *L* represents the labor, as measured by the number of employees in urban units in the *China City Statistical Yearbook*; *E* represents energy consumption; *Y* represents the gross product of a city, derived from the *China City Statistical Yearbook* and the provincial statistical yearbooks; *B* represents PM_2.5_, derived from the real time air quality release system of the Ministry of Environmental Protection of China, that is the National Control Site Hour Overall Data. We first found the average daily concentration of PM_2.5_ per hour, then multiplied the hourly concentration of PM_2.5_ by 24 to get the daily concentration of PM_2.5_, and finally divided the total daily concentration of PM_2.5_ by 365 to get the annual concentration of PM_2.5_. *g* is direction vector; ${w}_{E}$, ${w}_{Y}$, and ${w}_{B}$ represent the city’s energy consumption, GDP, and the standardized weight vector for PM_2.5_, respectively. ${\beta }_{E}$, ${\beta }_{Y}$, and ${\beta }_{B}$ represent energy consumption, GDP, and level factor vector of PM_2.5_ in cities, respectively. In the constraint conditions represented by $s.t.$, ${Z}_{n}$ is the weight coefficient; *n* is the city; and ${\beta }_{E}^{* }$, ${\beta }_{Y}^{* }$, and ${\beta }_{B}^{* }$ are the energy consumption, GDP, and the inefficiency value of PM_2.5_, respectively.

The energy efficiency derived from the DEA method is generally considered as more scientific and reliable; however, its calculations must be supported by other socioeconomic data, such as the capital stock and labor. These data are not published by the statistical agencies of some cities and counties. Therefore, we could only provide energy efficiency data for 189 cities using the DEA.

Energy consumption per unit GDP has also been used to approximately represent energy efficiency by some studies, such as Bor^91^, Duro & Padilla^92^, Cheng *et al*.^93^, and Cheng *et al*.^94^. Lower energy consumption, which drives economic growth, is often considered to be more energy efficient. Accordingly, we used the energy consumption per unit GDP to estimate the energy efficiency of 336 cities and 2,489 counties in China.

## Data Records

**Fig. 2:**
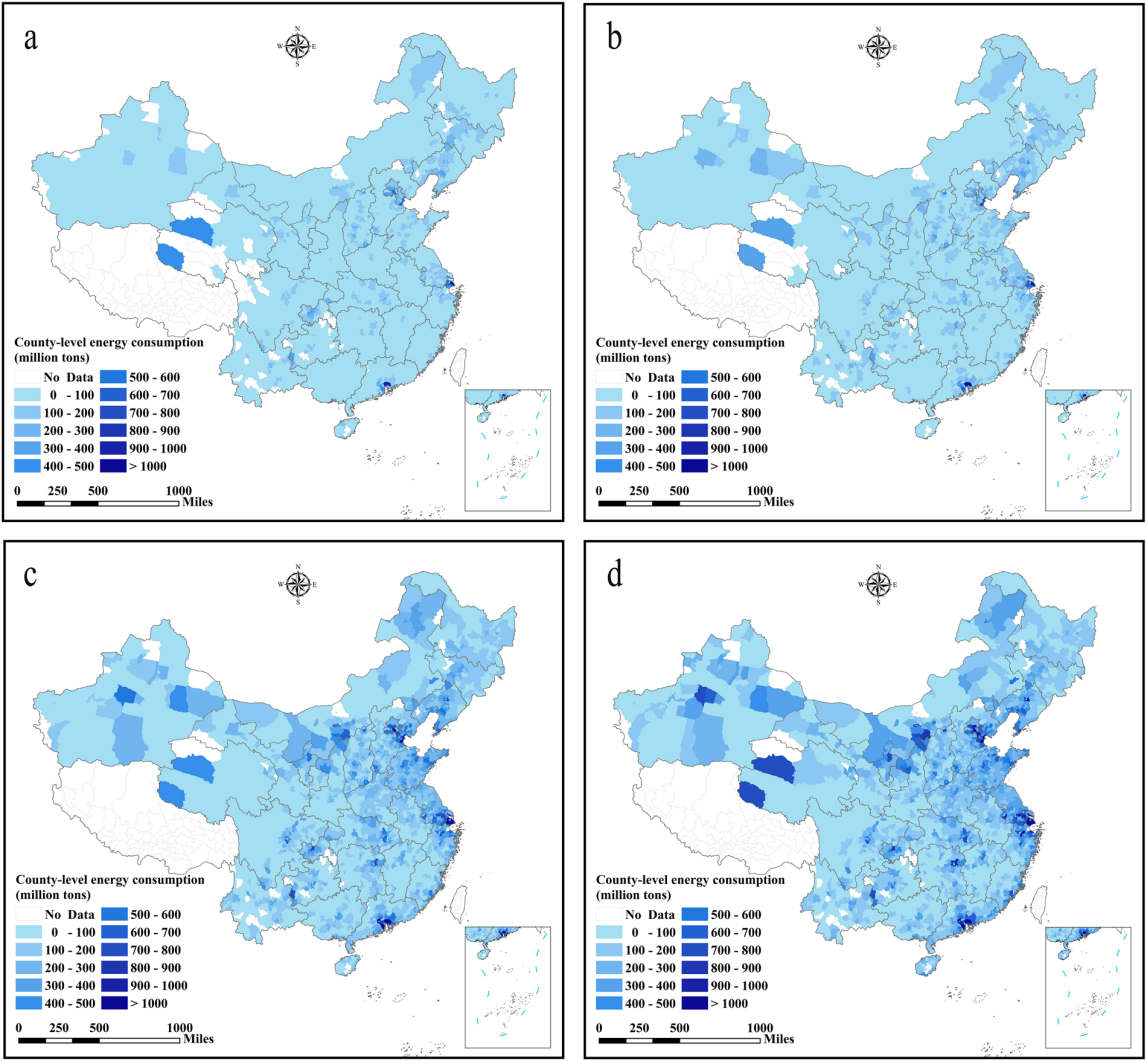
County-level energy consumption in China (unit: million tons of coal equivalents). (**a**) The energy consumption in 1997, (**b**) the energy consumption in 2003, (**c**) the energy consumption in 2010, (**d**) the energy consumption in 2017.

**Fig. 3:**
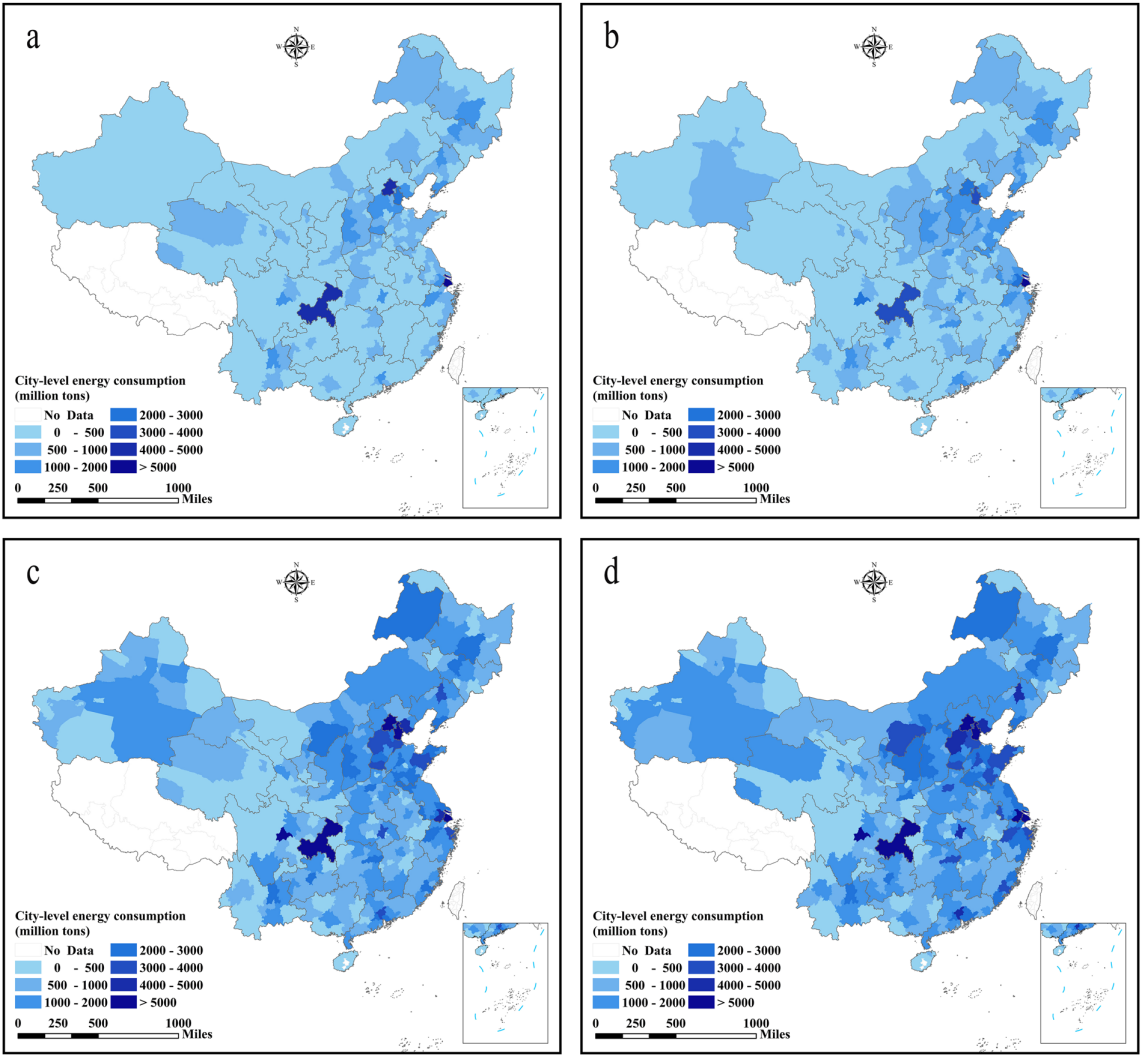
City-level energy consumption in China (unit: million tons of coal equivalents). (**a**) The energy consumption in 1997, (**b**) the energy consumption in 2003, (**c**) the energy consumption in 2010, (**d**) the energy consumption in 2017.

**Fig. 4:**
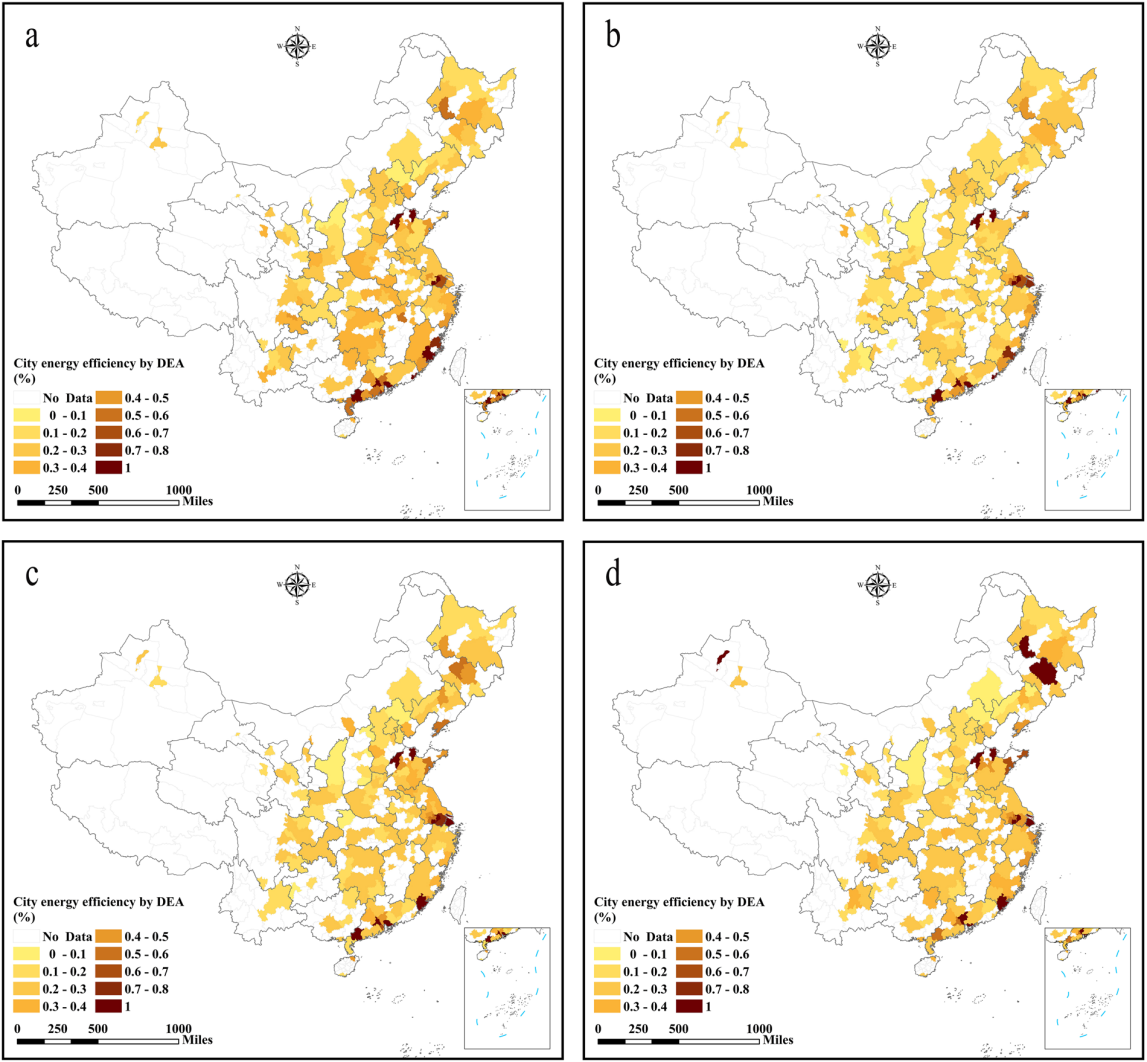
City-level energy efficiency in China obtained using DEA. (**a**) The energy efficiency in 2003, (**b**) the energy efficiency in 2007, (**c**) the energy efficiency in 2012, (**d**) the energy efficiency in 2016.

**Fig. 5:**
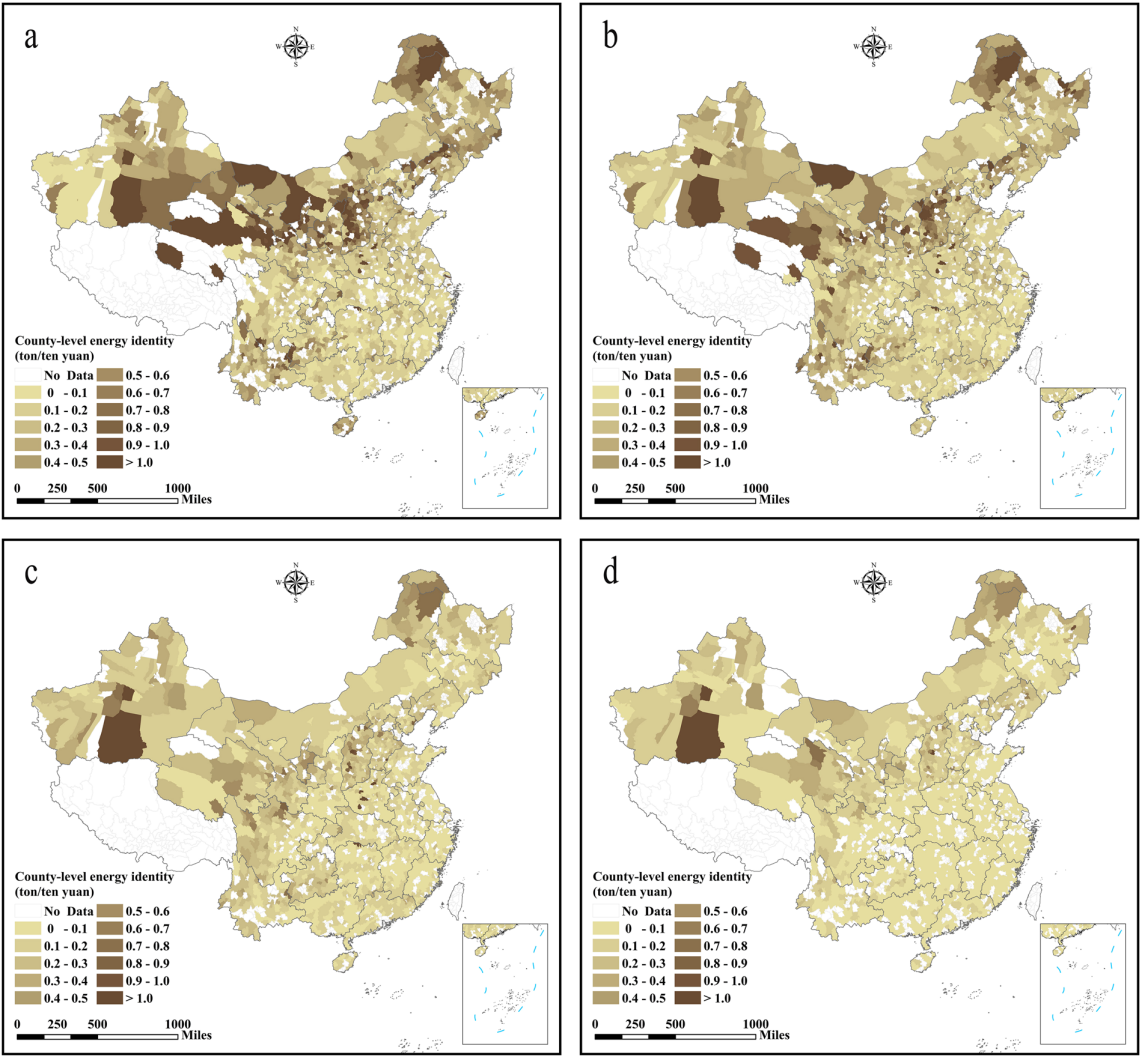
County-level energy efficiency in China obtained from energy consumption per unit GDP. (**a**) The energy efficiency in 1997, (**b**) the energy efficiency in 2003, (**c**) the energy efficiency in 2010, (**d**) the energy efficiency in 2017.

**Fig. 6:**
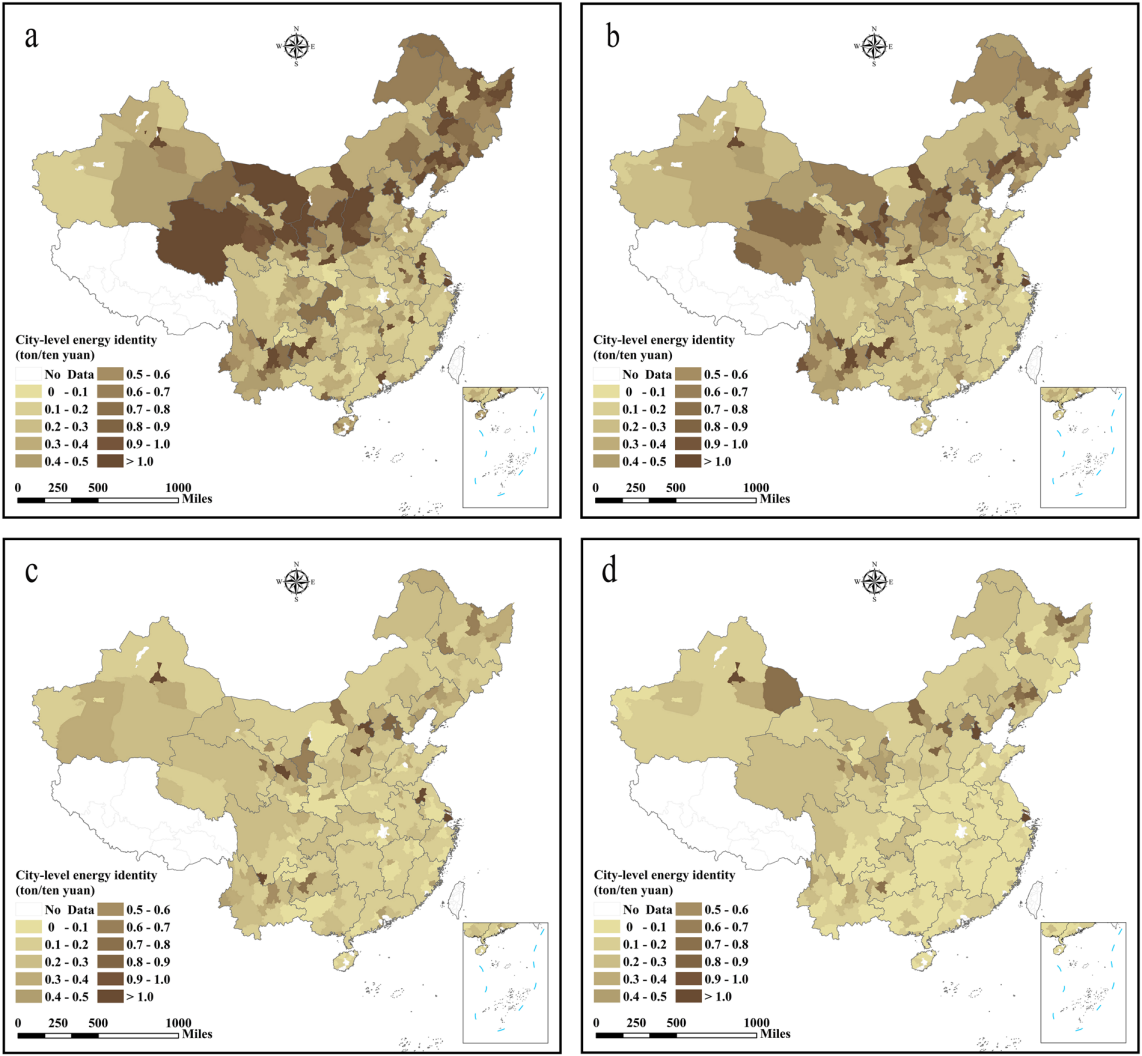
City-level energy efficiency in China obtained from energy consumption of per unit GDP. (**a**) The energy efficiency in 1997, (**b**) the energy efficiency in 2003, (**c**) the energy efficiency in 2010, (**d**) the energy efficiency in 2017.

This dataset provides a total of 6,085 data records (energy consumption and energy efficiency)^44^. These include: 336 city energy consumption data (1997–2017), 2,735 county energy consumption data (1997–2017), 189 city energy efficiency data (2003–2016) based on DEA, 336 city energy efficiency data (1997–2017) based on energy consumption per unit GDP, and 2,489 county energy efficiency data (1997–2017). The datasets and figures published herein are publicly available in the data files. Energy consumption data was measured in million tons of coal equivalents. The spatial and temporal distribution of energy consumption and energy efficiency at the city and county levels in China are shown in Figs. 2–6.

## Technical Validation

### Validity testing for energy consumption based on nighttime light data from satellites

**Fig. 7:**
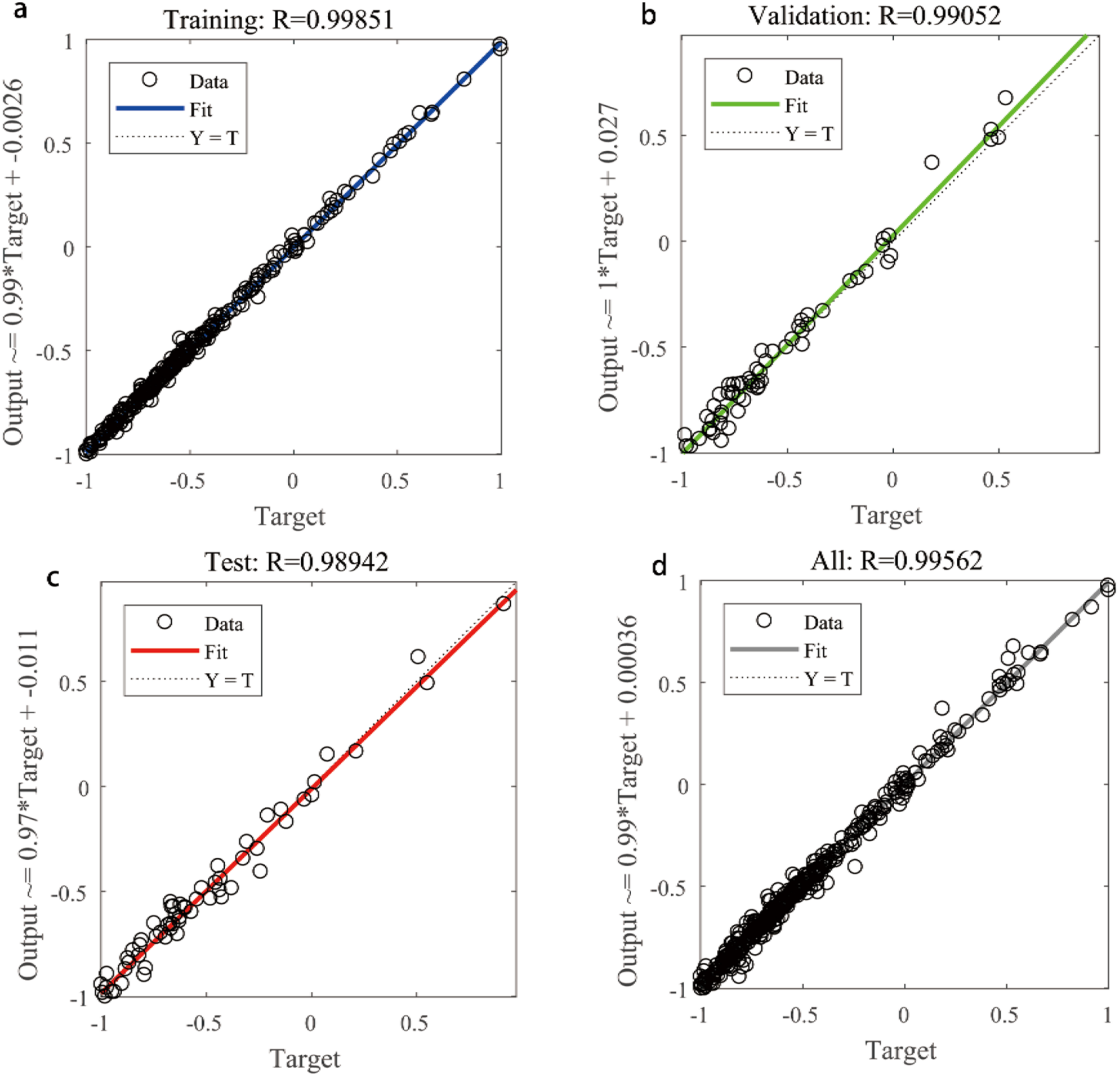
Test results for the mean pixel values in 2013. Data represent (**a**) the results with the supplementary input parameters based on the PSO-BP algorithm, (**b**) the results for only the mean pixel values of nighttime light values based on the PSO-BP algorithm, (**c**) the results with the supplementary input parameters based on the BP algorithm, and (**d**) the results with only the mean pixel values of nighttime light values based on the BP algorithm.

The fitting results of PSO-BP neural network algorithm are shown in Fig. 7. The coefficient of determination of the training set was 0.999, while that of the test and verification sets were 0.989 and 0.991, respectively. This shows that the model has good fitting effect and validity in the training, testing, and verification stages, and the overall coefficient of determination of the model was high at 0.9956, which is higher than that of Yue *et al*.^41^. In their work, the coefficient of determination using the econometrics estimation method was 0.734.

**Fig. 8:**
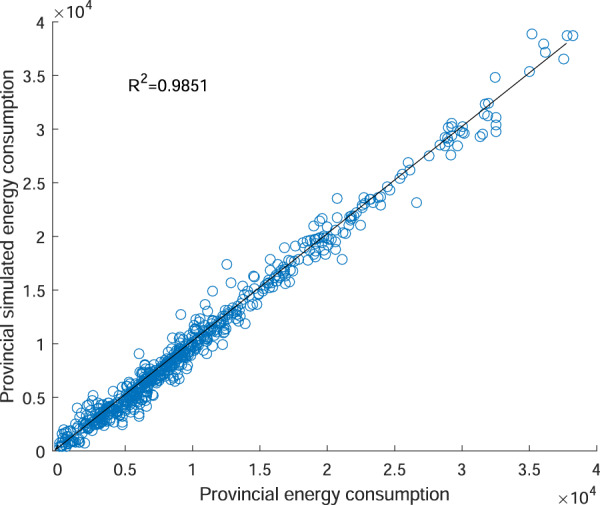
Training and test results for the relationship between provincial energy consumption and the sum of DN values of provincial night-light data (unit: million tons of coal equivalents).

Furthermore, we fit the county-level energy consumption data according to the province-level division by using the sum of the provincial simulated energy consumption, and the results were compared with official statistical provincial energy consumption data in the *China Energy Statistics Yearbook* from 1997 to 2017. The latter was published by the Chinese official agency, CNBS, and was used as the validation data for comparisons, as shown in Fig. 8. The provincial energy consumption data fitted in this study was highly correlated with the official statistical provincial energy consumption data, and the decisive coefficient was 0.9851. This shows that the energy consumption data obtained from fitting of satellite nighttime light data is reliable.

### Limitations and future work

There are three deficiencies in our datasets, which will provide future avenues for more accurate estimates of energy consumption and efficiency at the city and county levels in China. First, the city-and-county-level energy consumption data retrieved from satellite nighttime light data only comprises the total energy consumption data, and cannot identify the change of energy mix. Second, the provincial energy consumption data spliced using satellite nighttime light data do not include energy consumption data from land-use change and use of non-commercial biomass fuels, which results in underestimation of the energy consumption data of cities and counties. Third, considering the unavailability of socioeconomic data for some cities and all counties, such as labor and capital stock, even though data on energy consumption was provided for 336 cities and 2,735 counties from 1997 to 2017, the energy efficiency data based on the DEA included only 189 cities between 2003 and 2016.

Therefore, our future studies will focus on three aspects: First, in the retrieval of city-and-county-level energy consumption data, the disaggregated energy consumption data and other socioeconomic variables should be explored and introduced. Second, a provincial total energy consumption data covering more energy types should be obtained before comprehensively inverting the city and county energy consumption data through investigation. Third, the socioeconomic data of cities and counties should be obtained to support the use of DEA in evaluating the energy efficiency of all cities and counties in China.

## Usage Notes

The data of city-and-county-level energy consumption and efficiency provided in this study are valuable with practical applications in the field of energy economics, management, and policy, including the following: First, the provided data have the characteristics of wide spatial coverage and long time span. This unique panel-structured dataset can be used to observe the trajectories and spatial differences of energy consumption and efficiency on a micro-level than at national and provincial levels. Therefore, it can also be used to analyze the factors driving the changes and spatial differences of energy consumption and efficiency in cities and counties. Second, the panel-structured dataset of energy consumption and efficiency can be used to match other socioeconomic data at the city and county levels, and studies such as the economic effects of energy consumption, the environmental and social effects of energy consumption, and the coupling relationship between energy efficiency and economic development may be conducted. Third, the development of energy consumption and efficiency data at the city and county levels can not only contribute to the energy management at China’s grassroot-level governments, such as in the formulation and implementation of road maps for energy transition and energy efficiency improvement, but also provide a basis for the central and provincial governments to allocate the energy rights of cities and counties under the constraints of “carbon peak” and “carbon-neutral” targets. Fourth, the development of energy consumption and efficiency data at city and county levels could provide a more accurate assessment of the impact of the central government’s energy saving, emissions reduction, and low-carbon green policies, as well as other socioeconomic policies, for example, assessment of the impact of the “central heating,” “coal to electricity,” low-carbon pilot city, and carbon emissions trading right pilot policies on energy consumption and energy efficiency. Fifth, the method of retrieving micro-level energy consumption data by using satellite nighttime light data can also provide a reference for other developing countries and regions with limited energy statistics to evaluate their energy consumption and efficiency at the sub-national level.

## Supplementary information

Supplementary information

## Data Availability

MatLab (R2017b), STATA (16), and ArcGIS (10.5) are the major applications used to obtain the energy consumption and efficiency data. The code for PSO-BP matching the relationship between the two sets of satellite nighttime light data and the inversion of city and county energy consumption is provided in the Appendix. Codes and datasets for the DEA method, which measures energy efficiency in 189 cities, are also provided in the Appendix.
