# Supplementary material for: City- and county-level spatio-temporal energy consumption and efficiency datasets for China from 1997 to 2017
Source: Sci Data. 2022 Mar 24;9:101. doi: 10.1038/s41597-022-01240-6 (PMC8948207; doi:10.1038/s41597-022-01240-6)
Supplement: Supplementary file 1 — Supplementary information [file 41597_2022_1240_MOESM1_ESM.doc]

Supplementary information for

**City- and county-level spatio-temporal energy consumption and efficiency datasets for China from 1997 to 2017**

Jiandong Chen^1^, Jialu Liu^1^, Jie Qi^1^, Ming Gao^1^, Shulei Cheng1,[[1]](#footnote-2)*, Ke Li^2^, & Chong Xu^1^

^1^ *School of Public Administration, Southwestern University of Finance and Economics, Chengdu, Sichuan 611130, China*

^2^ *School of Statistics,, Southwestern University of Finance and Economics, Chengdu, Sichuan 611130, China*

The supplementary information provides additional content, including: (1) the codes of DEA method for energy efficiency; (2) the PSO-BP codes for matching the relationship among the provincial energy consumption and sum of DN values.

Number of pages: 6

# Contents

**S1.** Codes of DEA method for energy efficiency [Using STATA (16) to run]

**S2.** PSO-BP codes for matching the relationship among the provincial energy consumption and sum of DN values [Using MatLab (R2017b) to run]

**S1. Codes of DEA method for energy efficiency [Using STATA (16) to run]**

. net install ddfeff, from

(“https://raw.githubusercontent.com/kerrydu/ddfeff/master/”)

. matrix nweight=(0,0,1/3,1/3,1/3)

. gen ngk=0*capital

. gen ngl=0*labor

. gen gf=-1*energy

. gen ge=1*gdp

. gen gc=-1*pm25

nddfeff capital labor energy= gdp: pm25, dmu(area) time(year) wmat(nweight) gx(ngk ngl gf) gy(ge) gb(gc) sav(p2.dta,replace)

merge m:m area using p2.dta

drop _merge

gen eepi=[1-0.5*(B_energy+B_pm25)]/[1+B_gdp]

**S2. PSO-BP codes for matching the relationship among the provincial energy consumption and sum of DN values [Using MatLab (R2017b) to run]**

random_num = rand(1,630);

[value,index] = sort(random_num);

x_train = input(index(1:400),:)';

y_train = output(index(1:400));

x_test = input(index(401:630),:)';

y_test = output(index(401:630));

%Set the number of BPNN nodes

inputnum = 1;

hiddennum = 3;

outputnum = 1;

%Set the related parameters for PSO

sizepop = 10;% Population size

k = 50;% number of iterations

c1 = 2; c2 = 2;% learning factor

w = 0.8;% inertia factor

%Data normalization

[input_train,inputps] = mapminmax(x_train);

[output_train,outputps] = mapminmax(y_train);

%Determination of the number of optimization parameters

length = inputnum*hiddennum+hiddennum+hiddennum*outputnum+outputnum;

param = rand(sizepop,length);

speed = rand(sizepop,length);

%Establishing BPNN

net = newff(input_train,output_train,hiddennum);

%Initialization (individual best position lbest | global best position gbest | individual fitness fitness | group best fitness fitnessbest)

for i=1:sizepop

fitness(i,:) = func(param(1,:),inputnum,hiddennum,outputnum,net,input_train,output_train);

lbest(i,:) = param(i,:);

end

[value,index] = min(fitness);

gbest = param(index,:);% group extreme position (parameter)

fitnessbest = value;% group extreme fitness (minimum MSE)

% Parameter optimization

for T=1:k

for i=1:sizepop

speed(i,:) = w*speed(i,:)+c1*rand*(lbest(i,:)-param(i,:))+c2*rand*(gbest-param(i,:)) ;

param(i,:) = param(i,:)+speed(i,:);

fit = func(param(i,:),inputnum,hiddennum,outputnum,net,input_train,output_train);

if fit<fitness(i,:)

fitness(i,:) = fit;

lbest(i,:) = param(i,:);

end

if fit<fitnessbest

gbest = param(i,:);

fitnessbest = fit;

end

end

MSE(T,:) = fitnessbest;

end

% Build a model and predict (gbest is the best parameter)

w1 = gbest(1:inputnum*hiddennum);

b1 = gbest(inputnum*hiddennum+1:inputnum*hiddennum+hiddennum);

w2 = gbest(inputnum*hiddennum+hiddennum+1:inputnum*hiddennum+hiddennum+hiddennum*outputnum);

b2 = gbest(inputnum*hiddennum+hiddennum+hiddennum*outputnum+1:inputnum*hiddennum+hiddennum+hiddennum*outputnum+outputnum);

net.iw{1,1} = reshape(w1,hiddennum,inputnum);

net.lw{2,1} = reshape(w2,outputnum,hiddennum);

net.b{1} = reshape(b1,hiddennum,1);

net.b{2} = b2;

net.trainParam.epochs = 100;

net.trainParam.lr=0.1;

net.trainParam.goal=0.00001;

net = train(net,input_train,output_train)

1. ^^ Corresponding author: e-mail: chengsl@swufe.edu.cn. [↑](#footnote-ref-2)
